# Genetic variation in GST genes and urinary formic acid: a study in formaldehyde-exposed workers

**DOI:** 10.17179/excli2025-8955

**Published:** 2025-11-03

**Authors:** Reza Pourbabaki, Esmaeel Soleimani, Saeed Yousefinejad, Mostafa Saadat

**Affiliations:** 1 Student Research Committee, Shiraz University of Medical Sciences, Shiraz, Iran; 2 Department of Occupational Health, Shiraz University of Medical Sciences, Shiraz, Iran; 3 Department of Biology, School of Sciences, Shiraz University, Shiraz, Iran

## ⁯⁯

Formaldehyde, a colorless, flammable gas with a strong odor, belongs to the family of volatile organic compounds and is a common environmental and occupational contaminant. Workers in pathology laboratories and formaldehyde-producing industries are typically exposed to higher levels than the general population. Formic acid is a metabolite of formaldehyde excreted in urine (Chiarella et al., 2016[2]). One of the possible mechanisms underlying formaldehyde-induced toxicity is the production of reactive oxygen species, which subsequently induces oxidative stress.

Biological evolution has led to the development of detoxification pathways to minimize the effects of environmental exposures. Genetic variations in these pathways can affect their efficiency. A key example is the glutathione S-transferase (GST) superfamily of enzymes, which plays a critical role in detoxifying a wide spectrum of xenobiotics and protecting cells against oxidative damage. *GSTT1* and *GSTM1* are two members of this superfamily. In human populations, certain alleles result from the complete deletion of these genes. Individuals homozygous for a null allele at either locus lack the respective enzyme activity and are characterized as having a null genotype (Aloke et al., 2024[1]).

The reliability of urinary formic acid as a biomarker for monitoring inhalational formaldehyde exposure is controversial (Chiarella et al., 2016[2]), largely due to considerable inter-individual variation in its average baseline urinary level. Therefore, the aim of the present study was to investigate the relationship between the null genotypes of *GSTT1* and *GSTM1* and urinary formic acid levels in individuals exposed to formaldehyde.

This cross-sectional study involved 46 participants occupationally exposed to formaldehyde, comprising 7 individuals from pathology laboratories and 39 from a formaldehyde manufacturing plant in Fars province (Iran). All participants were Caucasians Muslims. Prior to enrollment, all participants were fully informed of the study objectives and provided written informed consent. The study was approved by the University Ethics Committee (IR.SUMS. SCHEANUT.REC.1402.094) and complied with the revised guidelines of the Helsinki Declaration (2000). Individuals with a history of smoking, alcohol consumption, or pre-existing kidney or liver diseases were excluded.

Air sampling was conducted within the breathing zone of each worker. Individual formaldehyde exposure was evaluated using NIOSH Method 3500 (Eller and Cassinelli, 1994[3]). Urine specimens were collected in the morning after two hours of exposure (Rauscher, 2022[5]), following analytical procedures described by Peteffi et al. (2016[4]). Genotyping was performed as described by Saadat and Saadat (2001[6]). The null genotype was defined by the absence of PCR product, and reaction validity was confirmed by amplification of a β-globin control.

Our participants were exposed to high levels of formaldehyde (0.772±0.573 ppm). The mean (± Standard Deviation) urinary formic acid concentration was 54.1±11.05 µg/mL. Normality of the distributions of formaldehyde and formic acid concentrations was evaluated with the one-sample Kolmogorov-Smirnov test. Given their non-normal distribution, the values were transformed to achieve a normal or near-normal distribution (Supplementary information, Table S1) for subsequent analysis. A significant correlation was observed between exposure to log-transformed formaldehyde and inverse-transformed urinary formic acid levels (r=-0.569, df=44, *p*<0.001).

A multivariable linear regression analysis was used. Based on this analysis, using log-formaldehyde and the number of null genotypes as predictor variables, a significant model was generated (see Table S2, F=14.4; df=2, 43; *p*<0.001; adjusted r²=0.373). In the fitted model, the partial correlation coefficient for the number of null genotypes showed a negative association with log-formic acid levels (partial r=-0.607, *p*=0.023). This indicates that the concentration of urinary formic acid was highest in participants with two active genes compared to those with one or two null genotypes.

The significant association between urinary formic acid levels and the number of null genotypes, along with the increase in the adjusted r² value (from 0.308 to 0.373) when *GSTT1* and *GSTM1* genotypes were considered, not only supports the existing controversy regarding the use of urinary formic acid as a biomarker but also confirms the key role of genetic predisposition in formaldehyde metabolism.

The association of the null genotypes of *GSTM1* and *GSTT1* with formaldehyde metabolism has important implications for occupational health risk assessments. Individuals with these null genotypes may be at increased risk of toxicity from formaldehyde exposure due to an impaired detoxification capacity. 

## Declaration

### Acknowledgments

The authors would like to thank the employers and employees for their cooperation.

### Artificial Intelligence (AI) - Assisted Technology 

The authors did not use artificial intelligence-based technologies, except for some English language corrections using “DeepSeek AI'”.

### Funding

This work was supported by the Shiraz University of Medical Sciences [Grant No. 28024].

### Competing interests

The authors declare that they have no conflict of interest.

### Availability of data and materials

The raw data are presented in the supplementary file.

## Supplementary Material

Supplementary information

## References

[1] Aloke C, Onisuru OO, Achilonu I (2024). Glutathione S-transferase: A versatile and dynamic enzyme. Biochem Biophys Res Commun.

[2] Chiarella P, Tranfo G, Pigini D, Carbonari D (2016). Is it possible to use biomonitoring for the quantitative assessment of formaldehyde occupational exposure?. Biomark Med.

[3] Eller PM, Cassinelli M (1994). NIOSH manual of analytical methods, US Department of Health and Human Services. Public Health Service, Centers for Diseases, Control and Prevention.

[4] Peteffi GP, da Silva LB, Antunes MV, Wilhelm C, Valandro ET, Glaeser J (2016). Evaluation of genotoxicity in workers exposed to low levels of formaldehyde in a furniture manufacturing facility. Toxicol Ind Health.

[5] Rauscher P (2022). American Conference of Governmental Industrial Hygienists (ACGIH) organisational update (March 2022). Occupational Health Southern Africa.

[6] Saadat I, Saadat M (2001). Glutathione S-transferase M1 and T1 null genotypes and the risk of gastric and colorectal cancers. Cancer Lett.

