# Supplementary material for: Genetic variation in GST genes and urinary formic acid: a study in formaldehyde-exposed workers
Source: EXCLI J. 2025 Nov 3;24:1606–8. doi: 10.17179/excli2025-8955 (PMC12876784; doi:10.17179/excli2025-8955)
Supplement: Supplementary information [file EXCLI-24-1606-s-001.pdf]

EXCLI Journal 2025;24:1606-1608 – ISSN 1611-2156
Received: September 24, 2025, accepted: September 30, 2025, published: November 03, 2025

Supplementary information to:

Letter to the editor:

GENETIC VARIATION IN GST GENES AND URINARY FORMIC ACID: A STUDY IN FORMALDEHYDE-EXPOSED WORKERS

Reza Pourbabaki[1,2], Esmaeel Soleimani[2], Saeed Yousefinejad[2], Mostafa Saadat[3,*]

1 Student Research Committee, Shiraz University of Medical Sciences, Shiraz, Iran
2 Department of Occupational Health, Shiraz University of Medical Sciences, Shiraz, Iran
3 Department of Biology, School of Sciences, Shiraz University, Shiraz, Iran

* **Corresponding author:** Mostafa Saadat, Department of Biology, School of Sciences, Shiraz University, Shiraz 71467-13565, Iran. Fax: +98-71-32280926; E-mail: saadat@shirazu.ac.ir

https://dx.doi.org/10.17179/excli2025-8955

This is an Open Access article distributed under the terms of the Creative Commons Attribution License (https://creativecommons.org/licenses/by/4.0/).

EXCLI Journal 2025;24:1606-1608 – ISSN 1611-2156
Received: September 24, 2025, accepted: September 30, 2025, published: November 03, 2025

Supplementary data:

Table S1: Normality assessment of study variables using the Kolmogorov-Smirnov test

	Formaldehyde (0 m)	Formic acid (µg/mL)	Log-transformed Formaldehyde	Inverse-transformed Formic acid
**Sample size (n)**	46	46	46	46
**Mean**	0.7722	54.16	-0.2620	0.0190
**Standard Deviation**	0.5734	11.05	0.3913	0.0028
**Test Statistic**	0.242	0.209	0.191	0.132
**Asymmetry probability (2-tailed)**	3.1×10^{-7}	2.7×10^{-5}	2.1×10^{-4}	4.3×10^{-2}

Table S2: Multivariable linear regressions analysis for association between Inverse-transformed urinary formic acid as dependent variable with log-transformed formaldehyde and number of null-genotypes of GSTs (as predictor variables)

Variables	Unstandardized coefficients		Standardized coefficients beta	Partial correlations	t	*p*
	B	Std. Error				
**Constant**	0.016	0.001			19.43	<0.001
Log-transformed formaldehyde	-0.004	0.001	-0.594	-0.607	-5.01	<0.001
Number of null-genotypes of GSTs	0.001	0.001	0.280	0.339	2.36	0.023

Note: The model was significant with F=14.40; df=2, 43; p<0.001; adjusted R^2=0.373.

EXCLI Journal 2025;24:1606-1608 – ISSN 1611-2156
Received: September 24, 2025, accepted: September 30, 2025, published: November 03, 2025

Raw data

ID	Groups	Sex	Age (Year)	Height (cm)	Weight (Kg)	MBI (Kg/m^2)	Duration of exposure (Year)	FA (ppm)	FOA (µg/mL)	GSTM1	GSTT1	Number of null genotype
1	Pathology	Male	54	180	68	20.99	25	.074	45.98	Present	Null	1
2	Pathology	Female	40	150	55	24.44	3	.074	47.71	Present	Present	0
21	Pathology	Female	44	155	56	23.31	18	.198	51.75	Null	Present	1
22	Pathology	Female	47	176	70	22.60	22	.198	50.13	Null	Null	2
23	Pathology	Female	45	175	80	26.12	11	.198	52.95	Present	Present	0
24	Pathology	Female	53	163	63	23.71	31	.198	50.13	Null	Present	1
25	Pathology	Male	42	178	130	41.03	17	.198	51.75	Present	Present	0
26	Pathology	Male	38	178	87	27.46	12	.242	50.77	Present	Null	1
28	Pathology	Male	39	175	83	27.10	4	.242	64.24	Present	Present	0
29	Pathology	Female	37	167	60	21.51	11	.242	67.70	Present	Present	0
31	Pathology	Female	34	164	58	21.56	5	.242	49.15	Null	Present	1
32	Industry	Male	23	186	89	25.73	3	.277	47.00	Present	Present	0
33	Industry	Male	31	170	75	25.95	3	1.502	55.61	Null	null	2
34	Industry	Male	32	175	66	21.55	4	1.256	51.36	Null	null	2
36	Industry	Male	51	182	94	28.38	20	.277	43.94	Present	Null	1
38	Industry	Male	42	179	74	23.10	17	.751	48.12	Null	Null	2
40	Industry	Male	32	185	75	21.91	5	.277	45.95	Present	Present	0
41	Industry	Male	38	178	78	24.62	20	.277	45.87	Present	Present	0
42	Industry	Male	46	174	77	25.43	20	.751	51.59	Present	Present	0
43	Industry	Male	43	180	85	26.23	20	.277	44.27	Null	Present	1
44	Industry	Male	33	190	110	30.47	3	1.646	96.56	Present	Present	0
46	Industry	Male	38	170	70	24.22	3	1.646	95.32	Present	Present	0
47	Industry	Male	30	188	70	19.81	6	1.256	55.58	Present	Present	0
48	Industry	Male	25	186	85	24.57	4	1.256	55.39	Present	Present	0
49	Industry	Male	40	178	94	29.67	14	.399	47.22	Null	Null	2
50	Industry	Male	41	167	70	25.10	9	.399	47.77	Null	Null	2
52	Industry	Male	25	178	92	29.04	3	1.646	65.35	Null	Present	1
53	Industry	Male	39	177	64	20.43	3	1.646	74.39	Present	Present	0
71	Industry	Male	43	174	93	30.72	20	1.557	57.41	Null	Null	2
82	Industry	Male	47	180	73	22.53	19	.353	46.75	Null	Null	2
85	Industry	Male	37	176	80	25.83	12	1.502	55.39	Null	Null	2
86	Industry	Male	35	173	60	20.05	12	1.502	58.11	Present	Present	0
88	Industry	Male	41	175	70	22.86	8	.353	47.03	Present	Present	0

EXCLI Journal 2025;24:1606-1608 – ISSN 1611-2156
Received: September 24, 2025, accepted: September 30, 2025, published: November 03, 2025

90	Indus-try	Male	33	187	89	25.45	3	.399	47.98	Pre-sent	Null	1
91	Indus-try	Male	42	170	66	22.84	8	1.256	53.07	Null	Pre-sent	1
94	Indus-try	Male	28	190	96	26.59	3	1.256	53.46	Pre-sent	Pre-sent	0
95	Indus-try	Male	32	175	77	25.14	3	.751	49.84	Pre-sent	Pre-sent	0
96	Indus-try	Male	45	179	80	24.97	16	.751	50.19	Pre-sent	Pre-sent	0
97	Indus-try	Male	31	180	99	30.56	3	1.646	58.20	Null	Pre-sent	1
100	Indus-try	Male	35	182	102	30.79	10	.751	49.07	Pre-sent	Pre-sent	0
101	Indus-try	Male	33	180	80	24.69	3	.399	48.37	Pre-sent	Pre-sent	0
104	Indus-try	Male	50	175	60	19.59	23	.751	48.08	Null	Null	2
105	Indus-try	Male	42	171	65	22.23	10	1.557	59.45	Null	Pre-sent	1
106	Indus-try	Male	46	195	105	27.61	15	1.557	57.77	Null	Null	2
107	Indus-try	Male	28	176	92	29.70	5	1.256	53.77	Pre-sent	Pre-sent	0
116	Indus-try	Male	32	175	58	18.94	6	.277	44.12	Null	Pre-sent	1

Note: FA=formaldehyde; FOA= formic acid
